# *Didymella pinodella*: An Important Pea Root Rot Pathogen in France to Watch Out For?

**DOI:** 10.3390/jof10010044

**Published:** 2024-01-05

**Authors:** Adnan Šišić, Jelena Baćanović-Šišić, Fernanda M. Gamba, Maria R. Finckh

**Affiliations:** 1 Section of Ecological Plant Protection, University of Kassel, 37213 Witzenhausen, Germany; mfinckh@uni-kassel.de; 2 Section of Organic Plant Breeding and Agrobiodiversity, University of Kassel, 37213 Witzenhausen, Germany; jelenabacanovic@gmail.com; 3 Plant Protection Department, Faculty of Agronomy, University of the Oriental Republic of Uruguay (UDELAR), Research Station Dr. M.A. Cassinoni, Ruta 3 K 363, Paysandú 60000, Uruguay; fernandagambafuica@gmail.com

**Keywords:** pea and wheat species mixtures, pea and wheat pathogens, *Fusarium*, aggressiveness, cultivar resistance

## Abstract

Root rot pathogens restrict pea and wheat production globally. In the EU, pea and pea-based cereal mixtures are being promoted; however, root rot pathogen dynamics in such mixtures are poorly understood. Winter pea and wheat were grown either in pure stands or in mixtures in the field in western France, and the severity of root rot in pea, wheat, and their mixtures, as well as the key pathogens associated with these crops, were assessed. Disease severity was moderate in pea and low in wheat, with no effect of sowing pattern. *Didymella pinodella*, a previously unreported pathogen in the pea–root rot complex in France, emerged as the most dominant pathogen in pea. It also occurred in low frequencies in wheat. Subsequent greenhouse aggressiveness tests showed that ten of the commonly grown pea cultivars in France lack resistance to *D. pinodella*. Among the *Fusarium* spp. isolated, *F. avenaceum* was the most frequent, occurring at similar frequencies in pea and wheat. In conclusion, *D. pinodella* may be an important pea root rot pathogen in France and there is a lack of resistance in the tested pea cultivars. In addition, *F. avenaceum* is a shared pathogen of wheat and pea.

## 1. Introduction

Foot and root rots of pea and wheat are of great importance worldwide. They are caused by a multitude of fungal pathogens that usually co-occur as disease complexes [1,2,3,4]. Symptoms can occur throughout the season and mainly include damping off, foot and root necrosis, often accompanied with vascular tissue discolouration, and stunting, often causing significant yield losses, especially in wet years [2,4,5].

Several pathogens have been identified as the causal agents of foot and root rots in pea and wheat, some of which are shared between the two crops. Among the most common shared pathogens are *Rhizoctonia, Pythium,* and *Fusarium* species, including *F. avenaceum, F. culmorum, F. tricinctum, F. oxysporum,* and *F. graminearum* [2,3,6,7,8]. The populations of pathogens, i.e., the presence or absence and relative abundance of individual species, involved in the disease complex vary with geographical region and climatic conditions [6,7,9,10,11,12,13]. In addition to generalist pathogens, there are several specialist pathogens, such as *Aphanomyces euteiches* in pea and other legumes [7,14] and *Gaeumannomyces graminis* var. *tritici* in wheat and other small-grain cereals, that can cause severe yield losses [5]. 

In pea, *Didymella pinodella* (syn. *Phoma pinodella, Phoma medicaginis* var. *pinodella*) is an important pathogen causing foot and root rots across Canada [10,15] and Australia [16,17,18], as well as in several European countries, including Germany, Denmark, and Sweden, where it has been recognised as the major component of the pea–root rot complex [6,8,19,20]. Previous reports have highlighted the aggressive nature of *D. pinodella* in pea [17,21,22,23] and demonstrated its capacity to asymptomatically colonise wheat roots, resulting in biomass reduction [22]. This fungal pathogen causes diseases in various other legume crops, including lentils, chickpeas, and faba beans [11,24]; however, to date, *D. pinodella* has not been reported in France as a root rot pathogen, where it has mostly been implicated as a minor part of the pea–Ascochyta blight complex, which affects the leaves, stems, and pods [25]. 

This study was initiated to investigate the effects of pea and wheat mixtures compared to their pure stands on the severity of root rot and to characterise the populations of *Fusarium* and *Didymella* species associated with the roots of these crops. Given the emergence of *D. pinodella* as the predominant pathogen in the pea–root rot complex, the study objectives were extended to assess its aggressiveness and evaluate the susceptibility of important French pea cultivars to this pathogen.

## 2. Materials and Methods

### 2.1. Field Experiment

A full factorial experiment was conducted in a randomised complete block design with three replicates in the growing season of 2018/2019 at the experimental station of the France National Research Institute for Agriculture, Food and Environment (INRAE)—Institute for Genetics, Environment and Plant Protection (IGEPP), Rennes, western France. The experiment included the winter wheat cultivar ‘Cellule’ and the winter pea cultivar ‘Aviron’, grown either in pure stands, together as random mixtures, or in alternate rows. In mixed plantings, wheat was sown at 30% of the standard rate of 350–400 seeds m^−2^, whereas pea was sown at the recommended rate of 100% (90 seeds/m^2^). Seeds were planted at a depth of 3–4 cm in mid-October in 3 × 6 m plots, with a row spacing of 25 cm. The field experiment was initially planned for three consecutive growing seasons; however, sample collection was possible only in 2018/19. In the following season (2019/20), the experiment failed due to unfavourable weather conditions, and, in the 2020/21 sowing season, the experiment had to be cancelled due to the COVID-19 pandemic. 

Foot and root rot disease severity was evaluated in 10 pea plants and 30 wheat tillers chosen randomly and dug up from each plot at the full flowering of pea. Pea root rot disease severity was visually assessed on a 0–8 scale (0 = healthy; 8 = dying plant) as described previously [12]. Disease severity classification for pea was as follows: no symptoms = plants with a score of 0, low = scores of 1–2, moderate = scores of 3–5, and high = scores of 6–8. The severity of foot rot symptoms in wheat was rated on a 0–3 scale according to Bockmann [26], with no symptoms = a score of 0; low = a score of 1, plants with lesions covering less than half of the stem circumference; moderate = a score of 2, plants with lesions spanning from 50% to 100% of the stem circumference; and high = a score of 3, plants with a rotten/broken stem [26]. There were no signs of any plants that were dying at the time of sampling. 

### 2.2. Pathogen Isolations from Field Grown Plants and Morphlogical Identifications of Collected Isolates

A subset of six pea and six wheat plants from each plot were used for fungal isolation and morphological identification following the methods described in Šišić et al. [11]. Briefly, roots were surface sterilised for 10 s with 3% sodium hypochlorite, rinsed in distilled water and placed on filter paper under a laminar flow hood to dry. Three approximately 1 cm long pieces per plant, representing the root, crown, and transition zone, were placed on Coons’ agar [27]. Roots included lateral and tap roots up to the point of seed attachment. The crown was regarded as the point of seed attachment up to approximately 0.5 cm below the soil surface, and the transition zone included the next ca. 1.5 cm up towards the stem [11]. Plates were incubated for 2 weeks at 20 °C under 12 h cycles of blacklight blue fluorescent light and dark. Plates were examined for *Didymella*-like colonies, which were selected based on morphology and the production of pycnidia and/or the presence of chlamydospores [28]. Simultaneously, *Fusarium*-like colonies were selected based on colony morphology, pigmentation, and/or the presence of fusoid conidia [29]. Subsequently, both *Didymella*-like and *Fusarium*-like colonies were subcultured onto half-strength potato dextrose agar (19 g/L Difco PDA and 10 g/L agar Sigma Aldrich, Steinheim, Germany). Colonies were incubated for an additional 2–3 weeks and purified via the transfer of single pycnidia (*Didymella* morphology) or hyphal tipping (*Fusarium* morphology). Morphological identifications were then conducted according to the methods described by Boerema et al. [28] for *Didymella* isolates and Leslie and Summerell [29] for *Fusarium* isolates. 

### 2.3. Molecular Validation of Fungal Species Identity and Phylogenetic Analyses

Three *D. pinodella*, four *F. avenaceum*, and nine *Fusarium oxysporum* species complex (FOSC) isolates were selected randomly and used to verify assigned morphological identity. Total genomic DNA was extracted from fresh mycelia collected from the fungal cultures actively growing on half-strength potato dextrose agar plates (19.5 g potato dextrose l-1 and 10 g agar l-1, Sigma Aldrich, Steinheim, Germany) following the protocol described by Sreelakshmi et al. [30]. The quantity and quality of DNAs were evaluated using a NanoDrop and stored in a TE buffer at −20 °C before PCR reactions. 

The identity of *D. pinodella* isolates was confirmed by sequencing portions of the β tubulin (tub2) gene region with the primers Btub2Fd and Btub4Rd [31]. The identity of *Fusarium* spp. was verified through amplification and sequencing portions of the translation elongation factor 1α (EF-1α) gene region using the primer pairs EF1 and EF2 [32]. Amplicons for each locus were generated following the protocols described in Šišić et al. [33]. Amplicons were visualised via electrophoresis on a 1% agarose gel and purified using the DNA Clean & Concentrator kit (Zymo Research, Freiburg, Germany) according to the manufacturer’s instructions. Sanger sequencing was performed at Macrogen Europe Laboratories (Amsterdam, Netherlands) in both directions using the same primer pairs used for the PCR amplifications. Obtained row sequence data were assembled in SeqMan Lasergene software version 7.1.0 (DNAStar, Madison, WI, USA). The resulting consensus sequences were compared with the National Center for Biotechnology Information (NCBI) [34] and FUSARIUM-ID v.3.0 [35] databases. Further validation of the taxonomic assignments of the isolates was performed via phylogenetic analyses.

**Figure 1:**
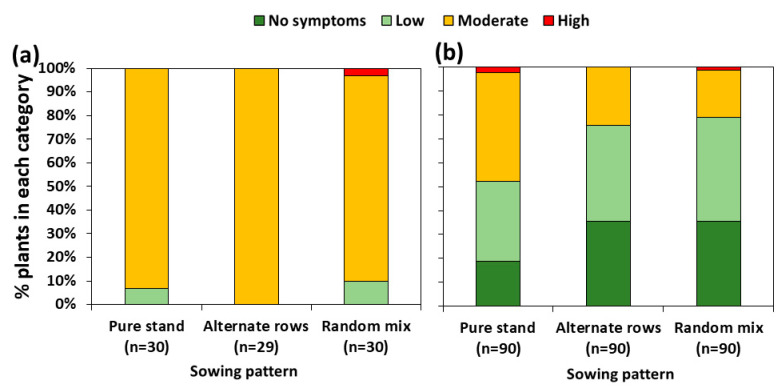
Effect of sowing patterns on the severity of foot and root rot symptoms of pea (**a**) and wheat (**b**). n = number of assessed plants per treatment. Reported *p*-values resulted from the Kruskal–Wallis test. Root rot disease severity expressed as follows: for pea plants: healthy–asymptomatic plants (plants with a disease assessment of score 0), low (plants with scores of 1–2), moderate (plants with scores of 3–5), and high (plants with scores of 6–8) disease severity [12]; for wheat plants: no symptoms = plants with a score of 0, low = plants with lesions covering less than half of their stems, moderate = plants with lesions spanning from 50% to 100% of the stem circumference, and high = plants with a rotten/broken stem [26].

The reference sequences used for *Didymella* phylogeny were selected based on the Chen et al. 2017 [36] study. The reference sequences used for *Fusarium* phylogeny comprised one representative strain of each species complex in the F2 *Fusarium* clade (see Figure 1 in the Geiser et al., 2021 study [37]). Additional pea-root-associated FOSC reference sequences were included in the analysis based on recent pea root rot surveys conducted in France [38] and the UK [39]. Alignments were generated using a MAFFT sequence alignment server (https://mafft.cbrc.jp/alignment/server/, accessed on 20 December 2023) [40,41] and manually edited in MEGA v. 6.06. [42]. Phylogenetic inference was based on maximum likelihood (ML). The ML analyses were performed with the online version of the IQ-TREE software available at http://www.cibiv.at/software/iqtree, accessed on 20 December 2023 [43]. The ModelFinder option was used to identify the optimal partitioning scheme and substitution models. The branch support in IQ-TREE was completed by the Shimodaira–Hasegawa-like approximate likelihood ratio test (SH-aLRT) and the ultrafast bootstrap (UFBoot) with 1000 replicates. The sequences generated in this study, along with the reference sequences and their GenBank accession numbers, are listed in Tables S1 and S2. 

### 2.4. Aggressiveness Tests in Pea

A greenhouse experiment was performed to test aggressiveness and to study the reaction of ten pea cultivars to *D. pinodella* isolates following methods previously described [22]. Of these, six cultivars were spring pea and four were winter pea, all provided by INRAE France and indicated as the most widely cultivated in France (Table 1).

Five *D. pinodella* isolates (FOEP 42.1500, FOEP 42.1501, FOEP 42.1503, FOEP 42.1027, and FOEP 42.1025) collected from symptomatic pea plants from the field experiment were grown on Coons’ agar for approximately 3 weeks under constant blacklight blue fluorescent light at 23 °C. Pycnidia and spores were collected from the agar surface using sterile microscope slides and approximately 15 mL of sterile distilled water. The suspension was passed through sterile cheesecloth to remove mycelial and pycnidial fragments, and spores were counted using a Fuchs Rosenthal hemocytometer (Paul Marienfeld GmbH & Co. KG, Lauda-Königshofen, Germany).

Two surface-sterilised pea seeds (70% ethanol for 5 min) were planted per 300 mL pot that contained approximately 400 g of autoclaved sand. Following sowing, inoculations were carried out with either three single *D. pinodella* isolates (FOEP 42.1500, FOEP 42.1501, or FOEP 42.1503) or an equal mixture of the five isolates listed above. Inoculation was carried out by drenching the sand with spore suspensions to achieve a 2 × 10^4^ spore g^−1^ substrate. Control pots were left non-inoculated and irrigated with distilled water. The experiment was arranged in a completely randomised design with four replicates for each pea cultivar. Pots were kept in the greenhouse at a 19 °C day and 16 °C night temperature, and a photoperiod of 16 h of light a day^−1^ (provided by 400 W high-pressure sodium lamps). Plants were watered daily with tap water. The number of surviving seedlings was recorded after 4 weeks. The plants were then removed from the pots, and the roots were separated from the above-ground parts, washed under running water, and evaluated for the severity of root rot symptoms and disease severity classes assigned as described above (Section 2.1). Above-ground fresh plant biomass was determined before drying at 105 °C until constant weight was achieved to determine dry plant biomass. In order to confirm that infection was a result of the inoculated pathogen, for each inoculated pea cultivar 4–6 roots were randomly selected, and *D. pinodella* isolates were re-isolated and identified morphologically using the protocol described above (Section 2.2.). 

### 2.5. Data Analysis

All data were analysed using the statistical software R version 4.3.0 [45]. The analysis of ordinal (semi-quantitative) disease severity rating data from field and greenhouse experiments was performed using the Kruskal–Wallis test in the agricolae package [46]. Sowing pattern (field experiment), isolate, and genotype (greenhouse experiment) were considered fixed effects. If significant treatment effects were observed (*p* < 0.05), mean ranking values were separated with the Kruskal multiple comparison test [46,47]. The analysis of the sowing pattern effects on the isolation frequencies of individual fungal species recovered from pea roots from the field experiment was performed on proportional data using generalised linear models (bayesglm) with a binomial distribution and logit link function [48]. Contrasts (*p* < 0.05) were employed to separate the factor levels using the LSmeans package [49]. In the greenhouse inoculation experiment, numerous plants failed to emerge in the inoculated treatments. To account for this, prior to the data analysis, disease severity scores of 8 (corresponding to a dead plant) were manually added to the level of the cultivars’ corresponding non-inoculated control. This approach allowed for the calculation of the percent emergence for each cultivar tested, allowing for natural variation in seed germination among cultivars.

## 3. Results

### 3.1. Health Status of Field-Grown Plants

Almost all pea plants showed moderate levels of root rot symptom severity (Figure 1a), exhibiting brown to black necrotic lesions on stems and tap roots that totally encircled the tissue. About one-third of the wheat tillers assessed showed no symptoms of diseases (Figure 1b), whereas the remaining plants exhibited mostly streaks of reddish-brown discolourations on lower stems (low symptom severity), which sometimes expanded to form lesions encircling approximately 50% of the stem (moderate symptom severity). There were no significant differences in root rot symptom severity among sowing treatments (*p* = 0.7 for pea, and *p* = 0.2 for wheat) (Figure 1).

### 3.2. Composition of Fungal Pathogens Associated with the Roots of Field-Grown Plants

A total of 102 *D. pinodella* isolates were isolated from pea, where about 93% of the pea plants were infected by this pathogen, which represented about 58% of all of the isolates recovered from this host (Table 2). The pathogen was recovered at higher frequencies (*p* = 0.02) from pea stems (~85% infection rate) than roots (~41%) and at intermediate frequencies from crown tissue (~63%). *Didymella pinodella* also occurred in wheat, but with much lower isolation frequencies (approx. 15% of the plants were infected), accounting for about 13% of the isolates recovered from wheat where it was mainly recovered from crown tissue (Table 2). There was no effect of sowing pattern on *D. pinodella* isolation frequencies (Table 3).

*Fusarium* spp. were isolated at similar rates from both hosts, i.e., approx. 55% of the isolates originated from pea and approx. 45% isolates originated from wheat. In total, a total of 137 *Fusarium* isolates were recovered, representing six species, among which *F. avenaceum* was the most common, accounting for ~53% of all *Fusarium* isolates collected. This pathogen infected ~41% of the pea plants and ~39% of the wheat plants, and was predominantly isolated from the stem and crown tissue of both crops (20–35%) (Table 2). The members of the *F. oxysporum* (FOSC) and *F. solani* (FSSC) species complexes combined accounted for an additional ~36% of the *Fusarium* isolates. Both species complexes were common in pea (FOSC = ~35% and FSSC = ~22% of infected plants), but less frequent in wheat (FOSC = ~15% and FOSC = ~7% of infected plants). In pea, *F. oxysporum* was more frequently (*p* = 0.02) isolated from roots (approx. 22%) compared to stems (~4%). The type of plant tissue from which isolations were made had no significant effect on the isolation frequency of *F. oxysporum* from wheat (*p* = 0.8) nor *F. solani* from both crops (*p* > 0.3) (Table 2). Most remaining species found, including *F. equiseti, F. crookwellense, F. dimerum,* and *D. pinodes,* were represented by only a few isolates. As for *D. pinodella*, there was no effect of sowing pattern on the isolation frequencies of any of the *Fusarium* species (Table 3). 

**Figure 2:**
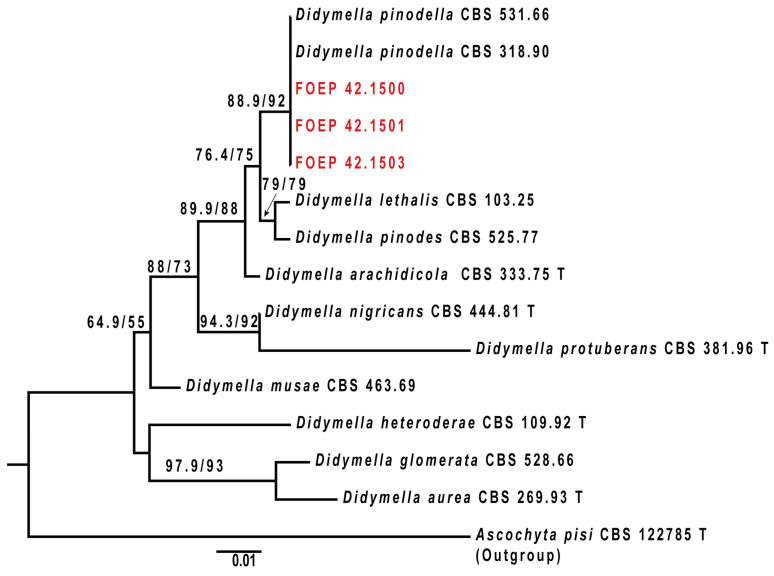
The maximum likelihood phylogenetic tree (IQ-TREE) inferred from the partial β tubulin (tub2) gene sequence alignments used to verify the identity of *D. pinodella* isolates generated in this study (designated as FOEP and highlighted in red). Epitype and ex-type strains are marked with a superscript ‘T’. Branch support values determined via a Shimodaira–Hasegawa-like approximate likelihood ratio test (SH-aLRT) and ultrafast bootstraps (UFBoot) are shown above branches. The scale bar indicates 0.01 expected changes per site. The tree is rooted to *Ascochyta pisi* (CBS 122785).

**Figure 3:**
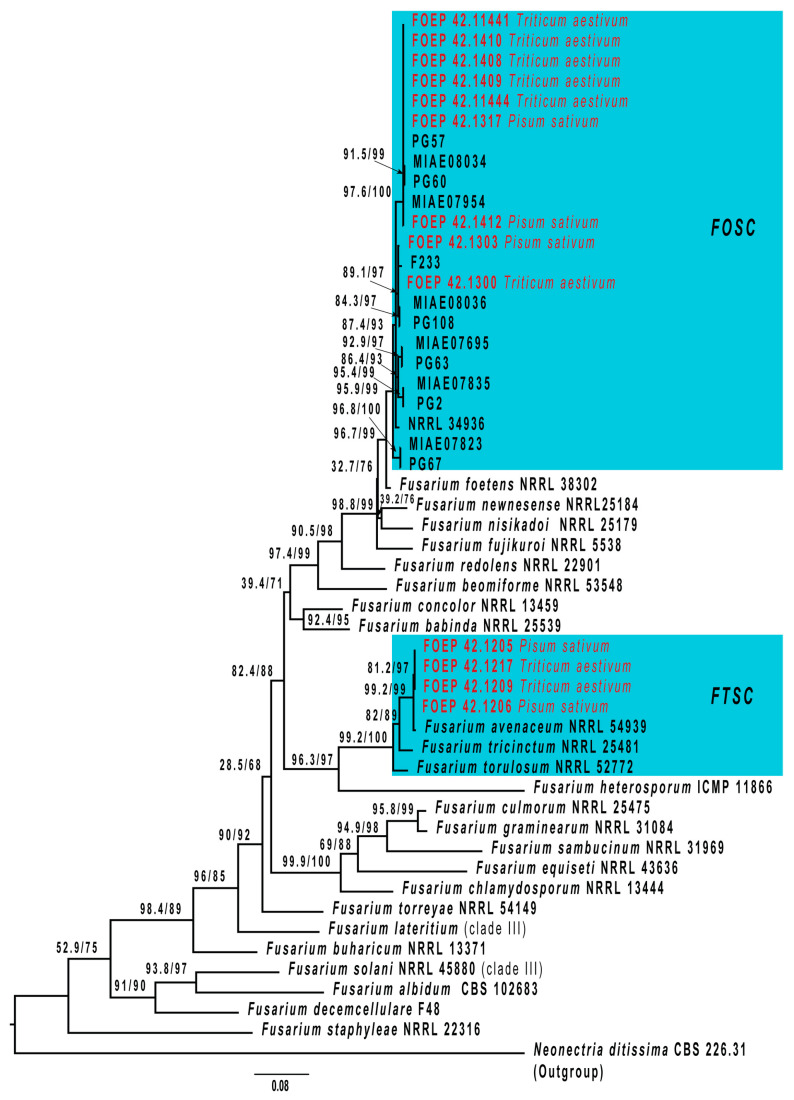
The maximum likelihood phylogenetic tree (IQ-TREE) inferred from the partial TEF1 alpha gene sequence alignments used to verify identity of *Fusarium* isolates. The isolates generated in this study (designated as FOEP and highlighted in red) were accommodated with the *Fusarium oxysporum* species complex (FOSC) and the *Fusarium tricinctum* species complex (FTSC), which are highlighted in turquoise. Branch support values determined via a Shimodaira–Hasegawa-like approximate likelihood ratio test (SH-aLRT) and ultrafast bootstraps (UFBoot) are shown above branches. The scale bar indicates 0.08 expected changes per site. The tree is rooted to *Neonectria ditissima* (CBS 226.31).

A phylogenetic analysis validated the morphologically assigned identifications of the isolates (Figure 2 and Figure 3). *Didymella pinodella* isolates matched *D. pinodella* CBS 531.66 and CBS 318.90 reference strains and were separated from the two sister species, *D. pinodes* and *D. lethalis* (Figure 2). Designated *F. avenaceum* isolates were placed in the *Fusarium tricinctum* species complex (FTSC), matching the *F. avenaceum* reference strain NRRL 54934. All FOSC isolates were accommodated in the *F. oxysporum* clade. A subgroup of eight isolates (three recovered from pea and five from wheat roots) closely matched the FOSC reference isolates MIAE08034, MIAE07954, PG57, and PG60, and two isolates (one recovered from pea and one from wheat roots) showed the closest genetic relationship with the *F. oxysporum* reference strains F233, PG108, and MIAI08036 (Figure 3). These reference isolates had previously been associated with diseased pea roots in France [26] and the UK [36]. 

### 3.3. Aggressiveness of Didymella pinodella to Pea in a Greenhouse 

**Figure 4:**
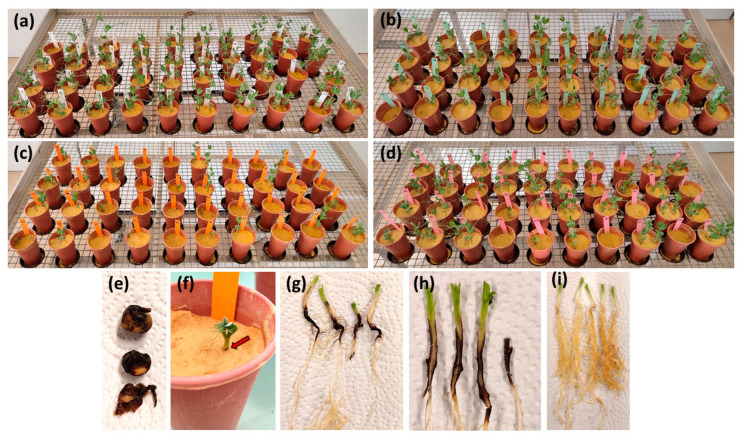
Pre-emergence death and disease symptoms on 10 pea cultivars following infection with *D. pinodella* isolates. (**a**) Non-inoculated control plants; (**b**) pea plants inoculated with *D. pinodella* isolate FOEP 42.1500; (**c**) pea plants inoculated with *D. pinodella* isolate FOEP 42.1503; (**d**) pea plants inoculated with the five-isolate mixture; (**e**) rotten pea seeds recovered at harvest; (**f**) stunted plant and blackening of the transition zone tissue in pea cv. Baccara following inoculation with isolate FOEP 42.1503; (**g**) black necrotic lesions on tap roots of pea cv. Safran concentrated around the zone of seed attachment, completely encircling the tissue following inoculation with isolate FOEP 42.1501; (**h**) cross-section of infected pea stems; and (**i**) healthy root system in non-inoculated control plants (pea cv. Baccara). The order of the 10 pea cultivars in pictures (**a**–**d**), vertically in pots from left to right: Orchestra, Kagnotte, Safran, Poseidon, Kaplan, Furious, Kayanne, Isard, Casini, and Baccara.

**Figure 5:**
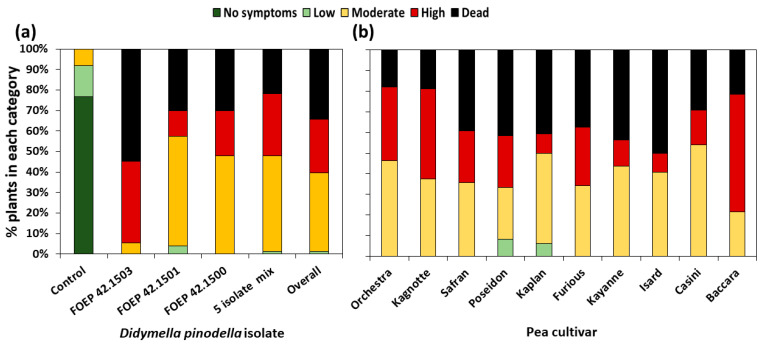
(**a**) Effects of *D. pinodella* isolates on pea emergence and root rot disease severity. Data are presented across 10 pea cultivars. (**b**) Mean reaction per pea cultivar to isolates of *D. pinodella*. Root rot disease severity expressed as healthy–no symptoms (plants with a disease assessment score of 0), low (plants with scores of 1–2), moderate (plants with scores of 3–5), and high (plants with scores of 6–7) disease severity. Dead (disease assessment score of 8) = pre-emergence death was calculated relative to the corresponding non-inoculated control.

All three *D. pinodella* isolates, as well as the mixture of five isolates, were pathogenic to pea, causing different levels of pre-emergence damping off and root rot severity (Figure 4 and Figure 5a). In data analysed across pea cultivars (overall isolate effects), FOEP 42.1503 was classified as the most aggressive (*p* < 0.001), causing a 55% reduction in seedling emergence (n = 40/73, i.e., 33 inoculated plants emerged relative to a total of 73 plants which emerged in control treatments). Of the remaining FOEP 42.1503 inoculated pea plants (i.e., those that did emerge), approximately 88% (n = 29/33) developed severe root rot symptoms, displaying stunted growth, black lesions on the root system, and/or the collapse of the entire taproot. Only about 12% (n = 4/33) of the emerged plants that had been inoculated with FOEP 42.1503 developed moderate levels of root rot, displaying necrotic lesions on tap roots that completely encircled the root system mainly in the area around the seed attachment (Figure 4 and Figure 5a). The effects of *D. pinodella* isolates FOEP 42.1500 and FOEP 42.1501, including the mixture of five isolates, were similar in terms of aggressiveness. These treatments were somewhat lower in aggressiveness compared to the most aggressive isolate, FOEP 42.1503, but were also classified as highly aggressive, causing a 22% to 30% reduction in plant emergence, severe root rot on 18–39% of the emerged plants, and moderate levels of root rot symptom severity on 60–76% of the emerged plants (Figure 5a).

**Figure 6:**
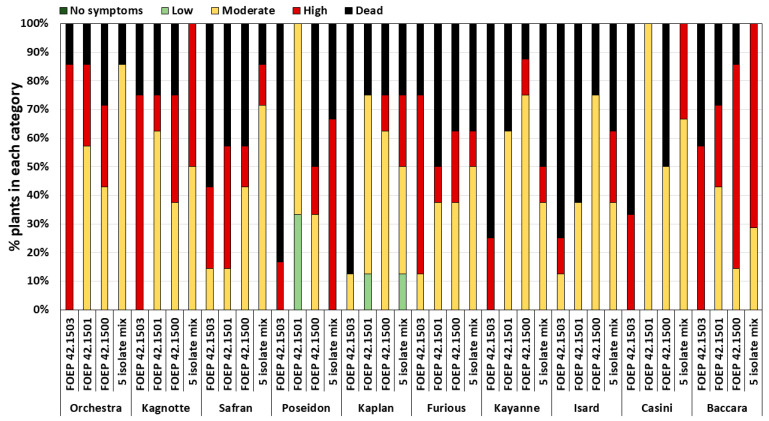
Reactions of 10 pea cultivars to three *D. pinodella* isolates (FOEP 1500, 1501, and 1503) and the mixture of 5 isolates. Root rot disease severity expressed as healthy–no symptoms (plants with a disease assessment score of 0), low (plants with scores of 1–2), moderate (plants with scores of 3–5), and high (plants with scores of 6–7) disease severity. Dead (disease assessment score of 8) = pre-emergence death was calculated relative to the corresponding non-inoculated control.

The pea cultivars (cvs.) used in this experiment showed some variability in their overall susceptibility, but these differences were not statistically significant (Figure 5b) (*p* = 0.4). Mean pre-emergence damping off ranged from 18% for cv. ‘Orchestra’ to 50% for cv. ‘Isard’ compared to the corresponding non-inoculated controls. Most of the remaining plants developed varying levels of root rot severity, ranging from moderate to high (Figure 5b). The reactions of individual cultivars indicated a wide range of susceptibility reactions and variations depending on the specific *D. pinodella* isolate used for inoculation (Figure 6). For example, FOEP 42.1503 caused an 83% reduction in plant emergence in the cv. ‘Poseidon’, and the effects of FOEP 42.1500 and the mixture of five isolates were intermediate (50% and 33% reduction in emergence, respectively), whereas the FOEP 42.1501 isolate was the least aggressive, causing no reduction in plant emergence and low to moderate root rot symptom severity in comparison to the corresponding non-inoculated control plants. By contrast, in the cv. ‘Safran’, isolates FOEP 42.1501 and FOEP 42.1503 caused severe root rot and pre-emergence damping off, the FOEP 42.1500 isolate effect was equally intermediate as for cv. ‘Poseidon’, and the mixture of five isolates was the least aggressive. Overall, none of the cultivars were resistant, with isolates causing varying levels of pre-emergence death and FOEP 42.1503 being consistently the most aggressive isolate in all pea cultivars (Figure 6). 

## 4. Discussion

In the current study, the severity of foot and root rot symptoms in field-grown plants was moderate in pea and low to moderate in wheat. There was no effect of sowing pattern on the root rot symptom severity or the isolation frequencies of any of the fungal species. *Didymella pinodella*, a previously unreported pathogen in the pea–root rot complex in France, was the predominant pathogen in pea, recovered from 93% of symptomatic roots accounting for 58% of the isolates recovered from this host. On wheat, this pathogen occurred only sporadically. The greenhouse tests indicate the lack of resistance in ten widely grown French pea cultivars to *D. pinodella* and the potential of this pathogen to cause significant yield reductions. *Fusarium* spp. were isolated at moderate rates from both pea and wheat with *F. avenaceum* predominant. They occurred at similar frequencies on both hosts highlighting their significance as shared pathogens. Other, mostly specialised, pathogens, including *F. oxysporum* and *F. solani,* were mainly recovered from pea, whereas most of the remaining *Fusarium* species were represented by a few isolates only. 

The emergence of *D. pinodella* at the research station in Rennes in western France as a predominant pathogen in the pea–root rot complex observed in our study on winter-grown peas contrasts the findings of a recent survey conducted in pea-growing areas across northern France on spring-grown green peas [38]. The authors reported that *Fusarium* spp. play a predominant role, whereas *D. pinodella* was not reported. One of the possible reasons for these contrasting results may be associated with the choice of agar media used to recover fungi from pea root pieces in the study of Gibert et al. [38]. As more than one single fungal species often colonise the roots simultaneously, the use of nutrient-rich malt extract agar for the culture-based fungal isolations, as employed by Gibert et al. [38], tends to favour fast-growing fungal species like *Fusarium* over slow-growing species such as *D. pinodella* [22,28]. We have found that using Coons’ agar [27] is particularly effective for isolating and identifying plant-associated *D. pinodella*. This medium promotes the growth of this pathogen and the formation of abundant pycnidia while also supporting the growth of *Fusarium* species. The agar choice as well as difficulties and often failure of cultural methods to recover other root pathogens, such as *A. euteiches* and *F. sporotrichioides/F. culmorum* in pea or *Verticillium dahliae* in chickpea, have been well documented [7,50,51,52]. It is also possible that the difference in the results between our study and Gibert et al. [38] could be attributed to site-specific agro-ecological effects, the type of pea, and/or the rotational history of the fields investigated. Gibert et al. [38] focused on spring green pea, which may have influenced the population of root-infecting fungi resulting in the reported prevalence of *Fusarium* species. Furthermore, in their study only 1 out of 22 sampled fields had a legume crop (i.e., common beans) in rotation 5 years prior to green pea sampling. Recent research has indicated, however, that an increase in *D. pinodella* abundance in the roots of pea and faba bean (*Vicia faba*) is linked to a greater frequency of these two [11,15,53] and likely other legume crops in rotation. The continued monitoring of this potentially important pea pathogen under field conditions is recommended.

The greenhouse data evaluating the resistance of widely grown pea cultivars in France, including both spring and winter varieties, to three *D. pinodella* isolates and the five-isolate mixture, indicated that all ten tested cultivars exhibited high susceptibility. Some variability in the reaction of individual pea cultivars to specific isolates was observed, with none of the cultivars showing high resistance to any of the isolates tested. All cultivars reacted with reduced emergence upon inoculation with at least one of the *D. pinodella* isolates. The emerged plants displayed moderate to severe root rot symptoms. Among individual isolates, the FOEP 42.1503 isolate was consistently the most aggressive. The symptoms caused by the remaining *D. pinodella* isolates, including the mixture of five isolates, were somewhat lower compared to the highly aggressive FOEP 42.1503 isolate and better corresponded to the symptoms observed in the field-grown plants. The ability of *D. pinodella* to cause severe root rot agrees with previous studies that also demonstrated the high pathogenicity of this pathogen to pea [22,54].

Taken together, the apparent dominance of *D. pinodella* in the pea–root rot complex observed in pea originating from northwestern France and the lack of resistance in tested pea cultivars warrants the need for additional research to monitor this pathogen in the major pea-growing areas of France. In order to overcome the limitations associated with culture-based fungal isolation, DNA-based detection techniques, such as quantitative real-time PCR assays, should be utilised [22,52,55]. 

The role of *F. avenaceum* in pea and wheat health is well documented. It is a serious and often highly aggressive pathogen in legumes and cereals [12,13,56,57], as well as a predominant species in the pea root rot complex in many regions of the world [6,7,58]. Our results suggest that *F. avenaceum* can become problematic in areas where legume and cereal crops are grown together in mixtures or in rotation as it can easily spread from one crop to the other. This pathogen may also play a role in the future expansion and development of important cereal disease complexes such as *Fusarium* head blight [59]. The members of the *F. oxysporum* and *F. solani* species complexes often show a high degree of host specificity, and both are well recognised as important pathogens of legumes but not cereals [11,29,57]. Both species complexes were reported as the most frequently detected in symptomatic spring pea root rots in 2017 across northern France [38]. The species *F. equiseti* has been previously shown to contribute to the reduction in diseases in various crops [60,61,62], including pea root rot caused by *D. pinodella* and *F. avenaceum* [21], the two most commonly isolated pathogens in this study. 

## 5. Conclusions

This study brings new information about the root rot complex of pea and wheat in France. It highlights, on the one hand, that winter- and spring-grown peas may harbour quite different pathogen communities and substantial differences with respect to the importance of *D. pinodella.* On the other hand, it underscores the importance of *F. avenaceum* as a shared pathogen in both crops. This may be of concern for French pea production, given the highly aggressive nature of *D. pinodella* coupled with the lack of resistance in the 10 pea cultivars tested in the greenhouse. Further research is thus recommended to monitor this pathogen in major pea-growing regions of France. Utilising DNA-based detection techniques, particularly quantitative real-time PCR assays [22], would enhance the precision of such monitoring efforts. Furthermore, the assessment of *D. pinodella* resistance in a larger set of pea cultivars representing the wider pea gene pool is recommended. 

## Figures and Tables

**Table 1:** Pea cultivars used in this study.

Cultivar Name	Cultivar Type ^1^
Orchestra	Spring
Kagnotte	Spring
Poseidon	Spring
Kaplan	Spring
Kayanne	Spring
Baccara	Spring
Safran	Winter
Isard	Winter
Casini	Winter
Furious	Winter

^1^ The pea cultivar descriptions are available at https://www.geves.fr/catalogue-france/; for the pea cv. ‘Baccara’, see Boutet et al., 2016 [44].

**Table 2:** Isolation frequencies of predominant pathogens recovered from pea and wheat stems, crowns, and roots collected from the field experiment conducted in the 2018/2019 growing season at the experimental station of the National Research Institute for Agriculture, Food and Environment (INRAE)—Institute for Genetics, Environment, and Plant Protection (IGEPP) in Rennes, France.

Crop	Tissue	n ^1^	*D. pinodella*	*F. avenaceum*	*F. oxysporum*	*F. solani*	*F. equiseti*
Pea	Stem	54	85.2 a	35.2 a	3.7 b	5.6	1.9
	Crown	54	63.0 ab	20.4 ab	14.8 ab	7.4	0.0
	Root	54	40.7 b	13.0 b	22.2 a	11.1	3.7
Total no. of isolates		177	102	37.0	22.0	13	3.0
% of plants affected			92.6	40.7	35.2	22.2	5.6
Wheat	Stem	54	1.9	25.9 ab	5.6	0.0	3.7
	Crown	54	13.0	31.5 a	7.4	1.9	9.3
	Root	54	1.9	9.3 b	5.6	5.6	9.3
Total no. of isolates		71	9	36	10	4	12
% of plants affected			14.8	38.9	14.8	7.4	18.5

^1^ n = total number of different pea and wheat plant parts used for isolations. For each crop separately, significant differences among means within a column are indicated by a different letter (generalised linear models with a binomial distribution and logit link function at *p* < 0.05, followed by Sa idak-adjusted LSMeans post hoc test). *D. pinodella* = *Didymella pinodella* (syn. *Phoma medicaginis* var. *pinodella*, *Peyronellaea pinodella*); *F* stands for *Fusarium,* e.g., *Fusarium avenaceum.* The isolation frequencies for the species *F. equiseti, F. crookwellense, F. dimerum,* and *D. pinodes* are not presented in the table due to their low isolation rates.

**Table 3:** Variations in isolation frequencies of the most common pathogens recovered from pea and wheat roots as affected by the sowing pattern.

Crop	Sowing Pattern	n ^1^	*D. pinodella*	*F. avenaceum*	*F. oxysporum*	*F. solani*	*F. equiseti*
Pea	Pure stand	18	83.3	33.3	27.8	22.2	5.6
	Alternate rows	18	100.0	55.6	38.9	16.7	11.1
	Full mix	18	94.4	33.3	38.9	27.8	0.0
*p*-value *			0.6	0.6	0.6	0.8	0.2
Wheat	Pure stand	18	11.1	27.8	11.1	0.0	5.6
	Alternate rows	18	16.7	55.6	27.8	16.7	33.3
	Full mix	18	16.7	33.3	5.6	5.6	16.7
*p*-value			0.9	0.7	0.2	0.2	0.2

*Didymella pinodella* syn. *Phoma pinodella*, *Phoma medicaginis* var. *pinodella*, and *Peyronellaea pinodella*). F stands for *Fusarium,* e.g., *Fusarium avenaceum*. ^1^ n = total number of plants used for isolations. * *p*-values used to examine the sowing pattern effects originate from generalised linear models (bayesglm), with a binomial distribution and logit link function performed for each pathogen separately.

## Data Availability

All relevant data are contained within the article and Supplementary Materials.

## Supplementary Materials

The following supporting information can be downloaded at: https://www.mdpi.com/article/10.3390/jof10010044/s1, Table S1: Genbank accession numbers for the β tubulin (tub2) gene region of the *Didymella pinodella* sequences generated in this study, along with the *Didymella* spp. reference strains and their GenBank accession numbers used to examine phylogenetic relationships among collected *Didymella* isolates. Table S2: Genbank accession numbers for the translation elongation factor 1α (EF-1α) gene region of the *Fusarium* sequences generated in this study, along with the *Fusarium* reference strains and their GenBank accession numbers used to examine phylogenetic relationships among collected *Fusarium* isolates.
